# Relationship Between Intraneural Hypervascularization of the Median Nerve and Electromyographic Severity Stages in the Diagnosis of Carpal Tunnel Syndrome

**DOI:** 10.7759/cureus.83951

**Published:** 2025-05-12

**Authors:** Fouzia Haddani, Fatima Zahrae Taik, Nihad Takhrifa, Imane Berrichi, Khadija Berrada, Hafida Majdaoui, Hicham Fadel, Rachid Belfkih, Fatima Ezzahra Abourazzak

**Affiliations:** 1 Rheumatology, Mohammed VI University Hospital, Faculty of Medicine and Pharmacy, Abdelmalek Essaadi University, Tangier, MAR; 2 Neurology, Mohammed VI University Hospital, Faculty of Medicine and Pharmacy, Abdelmalek Essaadi University, Tangier, MAR

**Keywords:** carpal tunnel syndrome, colour doppler, doppler, electroneuromyography, median nerve, neuromuscular ultrasound

## Abstract

Background: Ultrasound is increasingly playing a role in the diagnosis of carpal tunnel syndrome (CTS). The interest of the cross-sectional area (CSA) has been widely studied in assessing the severity of CTS. However, few studies have examined the usefulness of colour Doppler in evaluating the severity of CTS.

Objective: This study aims to evaluate the usefulness of colour Doppler ultrasound in assessing the severity of carpal tunnel syndrome compared to electroneuromyography (ENMG).

Methods: This is a cross-sectional study of patients over 18 years of age with typical signs of CTS, conducted over one year. All patients underwent ultrasound, including the measurement of the CSA at the entrance to the carpal tunnel, a colour Doppler, and an electroneuromyographic evaluation within a one-week interval. Using the Rosenbaum and Ochoa severity grading, patients were divided into four groups according to the stage of electrophysiological severity.

Results: Our study included 71 patients with typical signs of CTS, of whom 69 (97.2%) were female. The mean age of patients was 51.94 ± 10 years. CTS was bilateral in 54 patients, with each wrist considered an independent case. A total of 125 wrists were examined by ultrasound and ENMG. Colour Doppler was positive in 24 (19.2%) of the wrists studied. ENMG was normal in five (4%) wrists, 51 (40.8%) wrists were considered mild, 26 (20.8%) moderate, and 43 (34.4%) severe. Colour Doppler was positive in three (12.5%), three (12.5%), and 18 (75%) of the mild, moderate, and severe cases, respectively. None of the patients with normal ENMG had a positive colour Doppler. After analysis, there was a statistically significant difference between the non-severe and severe groups regarding Doppler positivity (p < 0.001).

Conclusion: Our study concludes that colour Doppler ultrasound can predict the severity of CTS, showing a difference between the severity of CTS and the presence of intraneural hypervascularisation of the median nerve in colour Doppler.

## Introduction

Carpal tunnel syndrome (CTS) is the most common peripheral neuropathy caused by compression of the median nerve in the carpal tunnel, a non-stretching osteofibrous tunnel. It affects 3.8% to 4.9% of the general population, with a higher prevalence in women [1].

The positive diagnosis of CTS is generally straightforward, based on clinical symptoms such as paraesthesia localised in the median nerve territory. These symptoms can radiate to the forearm or elbow and are often relieved by massaging or shaking the hand. Physical examination includes the Tinel's test (dysesthesia with percussion over the median nerve) and the Phalen's test (dysesthesia with wrist flexion). 

Electroneuromyography (ENMG) helps confirm the diagnosis in doubtful cases, especially when surgery is indicated. It also allows ruling out certain differential diagnoses, such as cervical radiculopathies, polyneuropathy, brachial plexopathies, or other forms of mononeuropathies, and it assesses the severity [2]. Evaluating the severity of CTS is essential for guiding treatment and must be systematically determined. However, ENMG has some limitations, including variable sensitivity and specificity, false negatives, and false positives [3-5]. These limitations suggest the need for complementary or alternative diagnostic methods that are less invasive and more cost-effective, such as sonography.

CTS is very common in occupations involving repetitive hand movements, pressure, or high force. It can be idiopathic or secondary to conditions such as endocrinopathy (including diabetes, hypothyroidism or acromegaly), rheumatism (mainly rheumatoid arthritis), amyloidosis, or local compression caused by flexor tenosynovitis, accessory muscles, a low muscular body insertion, a ganglion cyst, or a giant cell tumour of the tendon sheath. Identifying these causes is essential for guiding therapeutic management [6].

Ultrasound is a quick, harmless, accessible, and inexpensive examination. In addition to ENMG, it plays an increasingly important role in CTS diagnosis by allowing the study of the morphology of the median nerve, its anatomical relationships, and the identification of local causes in B-mode. It also quantifies intraneural and epineural blood flow in Doppler mode. Increased blood flow can be detected during nerve compression, chronic inflammatory polyneuropathy, neurolymphomatosis, or after nerve trauma [7-10]. This can be assessed using colour Doppler or power Doppler.

Ultrasound is increasingly used in diagnosing CTS, including assessing the median nerve cross-sectional area (CSA), hypoechogenicity, bowing of the flexor retinaculum, and flattening index. The interest of CSA has been widely studied in diagnosing CTS severity, showing a correlation between CSA and the positive diagnosis and severity of CTS [11-13]. However, few studies have examined the use of colour Doppler in diagnosing CTS severity.

Our work aims to evaluate the usefulness of colour Doppler ultrasound in diagnosing CTS severity compared to ENMG.

## Materials and methods

Study design

This was a cross-sectional study carried out over a period of one year, from January 2022 to January 2023, at the Rheumatology and Neurology Department of the Mohamed VI Tangier University Hospital in Tangier, Morocco. A free-and-informed-consent form was obtained from all participants, and the study was approved by the University Hospital Center, Ethics Committee of Tangier (CEHUT), under the approval number 01/2022.

Patients

Patients over 18 years of age presenting with paraesthesia in the median nerve territory were included in the study. Provocative tests (Phalen's and Tinel's tests), along with examinations of sensitivity and motor skills, were performed on all patients. Patients who had undergone corticosteroid infiltration or carpal tunnel surgery in the previous six months were excluded. Additionally, patients with peripheral neuropathy that could be confused with CTS, such as polyneuropathy, radiculopathy, proximal median nerve involvement, a history of wrist fracture, or previous surgical intervention on the wrist, were also excluded.

Clinical assessment

Data were collected on a pre-established form, from both the dominant and nondominant wrists, including demographic information (age, sex, and occupation), medical history, and clinical data. Pain intensity was assessed using the Visual Analogue Pain Scale (VAS). Tinel's and Phalen's tests were performed on all patients. Comorbidities, including diabetes, hypothyroidism, and rheumatoid arthritis, were also recorded.

Ultrasound assessment

All subjects were examined using B-mode ultrasound and colour Doppler with a MyLab X5 device from Esaote S.p.A (Genoa, Italy), equipped with a 21 Hertz linear probe. The ultrasound evaluations were conducted by two expert rheumatologists, and inter-observer variability was tested. All ultrasound examinations were performed with the patient in a seated position, forearm in supination, elbows bent at 90°, and wrist in a neutral position. The median nerve was examined in both transverse and longitudinal planes at the proximal part, immediately proximal to the flexor retinaculum, and the distal carpal tunnel, at the level of the trapezium and the hook of the hamatum.

The CSA of the median nerve was measured at the carpal tunnel inlet, identified based on the bony landmarks of the scaphoid and pisiform bones, using manual continuous boundary tracing, excluding the hyperechoic rim of the epineurium of the median nerve. A measurement above 10 sq mm was considered pathological. Echogenicity, flattening index, and anterior bulge of the retinaculum were assessed by ultrasound. The sign of the median nerve notch was sought, visualised on a longitudinal section, which corresponds to an abrupt disparity in the caliber of the median nerve, which is enlarged upstream of the stenosis and flattened within the canal. The vascularization of the median nerve was assessed by colour Doppler, it is considered positive when an intraneuronal or perineuronal spot is present. Median nerve mobility was dynamically assessed with finger flexion and extension, looking for bulging of the transverse carpal ligament and alteration of median nerve shape. Any anatomical abnormalities detected during the exam were noted.

ENMG

All patients underwent electromyographic examination within one week of the ultrasound. The electromyographic examination was performed by three neurologists, and inter-observer variability was tested. The severity of CTS was defined according to the Stevens Classification System as mild, moderate, and severe (Table 1) [14,15].

**Table 1:** Stevens Classification System: electrodiagnostic criteria for determining the severity of CTS NCS: nerve conduction study; SNAP: sensory nerve action potential; CAMP: compound muscle action potential; CTS: carpal tunnel syndrome Sources: [14, 15]

Electrophysiological severity of CTS	Stevens Classification System	
	Sensory NCS	Motor NCS
Mild (At least three of the sensory and motor nerve conduction classifications)	1. 14 cm wrist stimulation, peak latency >3.7 ms; 2. 14 cm wrist stimulation, peak latency: proximal 7 cm > distal 7cm; 3. Transcarpal 5cm short-segment latency: onset latency> 1.3 ms, peak latency> 1.5 ms; 4. 14 cm SNAP amplitude: 16-20 µV; 5. Conduction block greater than 50% in wrist palm stimulation if 14cm stimulation amplitude ≥ 20 µV	6. Distal latency >4.2ms; 7. CAMP amplitude: 4.1-4.5mV
Moderate (Mild plus at least two of the sensory and motor nerve conduction classifications)	1. Wrist stimulation (14 cm) SNAP amplitude≥ 6-15 µV; 2. Conduction block greater than 50% in wrist and palm stimulation if SNAP≥ 10µV with 14 cm wrist stimulation	3. CAMP amplitude: 2.1-4mV
Severe (Moderate plus one of the sensory and motor nerve conduction classifications)	1. SNAP amplitude ≤ 5 µV	2. CAMP amplitude ≤ 2mV

Statistical analysis

Continuous variables were expressed as mean ± standard deviation (mean ± SD) if the distribution was homogeneous or as median and interquartile range if the distribution was skewed. Categorical variables were reported as numbers and percentages. The Doppler comparison among the four groups was carried out using the chi-square test. If one of the theoretical numbers was less than five, the chi-square test between two groups (by grouping the categories) was used instead. The statistical significance level was set at p < 0.05. All analyses were performed using IBM SPSS Statistics software, version 21.0 (IBM Corp., Armonk, NY).

## Results

Demographic and clinical characteristics

The study included 71 patients with typical signs of CTS. The mean age of patients was 51.94±10 years, with a predominance of women (69, 97.2%). CTS was bilateral in 54 patients and unilateral in 17 patients, with each wrist considered an independent case. A total of 125 wrists were examined by ultrasound and ENMG. The symptom duration was 5.3 (0.10-24) years, and the mean pain VAS was 4.7±2. The sociodemographic and clinical characteristics are represented in Table 2.

**Table 2:** Sociodemographic and clinical characteristics of the study population **Values expressed as percentage (%); "Values expressed as mean±standard deviation; **Values expressed as median (interquartile range) CTS: carpal tunnel syndrome; VAS: Visual Analogue Scale

Number of patients (N)	N=71
CTS bilaterality*	
Unilateral	24
Bilateral	76
Gender*	
Female	97.2
Male	1.6
Age"	51.94±10
Duration of disease in years**	5.3 (0.10-24)
Pain VAS"	4.7±2
Tinnel test positivity*	84.8
Phalen test positivity*	83.2
Type of CTS*	
Idiopathic	74.4
Secondary	25.6
Comorbidity*	
Diabetes	24
Hypothyroidism	12
Rheumatoid arthritis	4.8

Ultrasound characteristics

**Figure 1:**
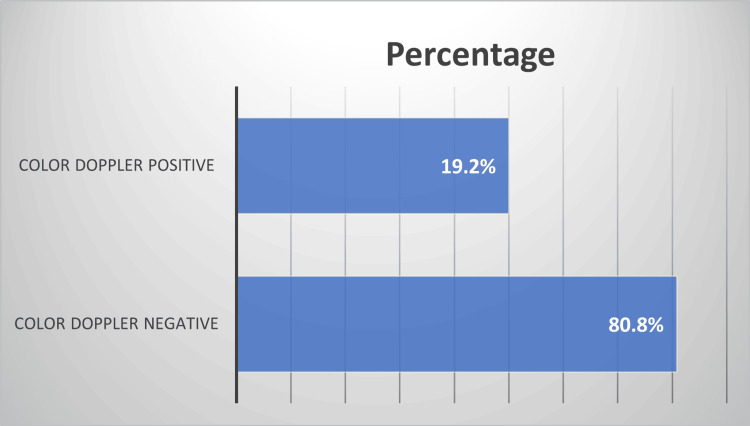
The percentage (%) of colour Doppler in patients with CTS CTS: carpal tunnel syndrome

**Figure 2:**
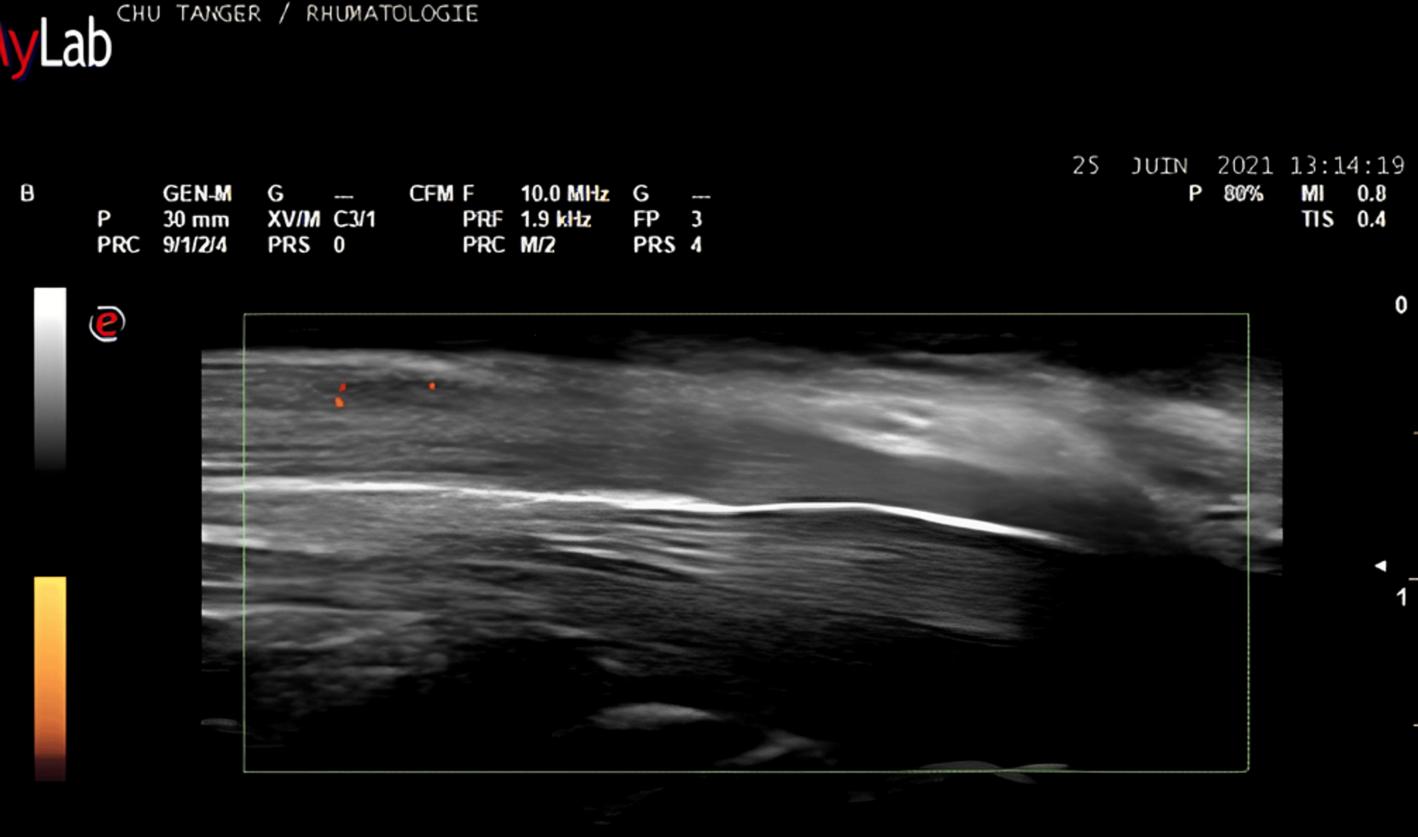
A longitudinal ultrasound image demonstrates intraneural vascularisation of the median nerve.

The mean CSA of the median nerve was 13 ± 5 sq mm. The ultrasound characteristics are represented in Table 3. Colour Doppler was positive in 24 (19.2%) of the wrists studied (Figure 1). ENMG was normal in five (4%) wrists, none of which had a positive colour Doppler. Of the wrists examined, 51 (40.8%) were considered mild, 26 (20.8%) moderate, and 43 (34.4%) severe. Colour Doppler was positive in three (12.5%) (Figure 2), three (12.5%), and 18 (75%) wrists, respectively (Table 4).

**Table 3:** Ultrasound characteristics of the study population *Values expressed as percentage (%); "Values expressed as mean±standard deviation CSA: cross-sectional area; N: number

Number of wrists	N = 125
CSA (sq mm) "	13 ± 5
Flattening index"	3.24 ± 0.9
Anterior bowing of flexor retinaculum (mm) "	2.6 ± 1.07
Positive notch sign*	16
Echogenicity*	
Hypoechogenicity	43.2
Normal	56.8

**Table 4:** Comparison between the colour Doppler and ENMG classes in the study population *Values expressed as percentage (%) N: number; CTS: carpal tunnel syndrome; ENMG: electroneuromyography

Severity	Normal ENMG	Mild CTS	Moderate CTS	Severe CTS	Total
N (*)	5 (4)	51(40.8)	26 (20.8)	43 (34.4)	125
Colour Doppler positive *	0	3 (12.5)	3 (12.5)	18 (75.0)	24
Colour Doppler negative *	5	48 (47.5)	23 (22.8)	25 (24.8)	101

Colour Doppler and ENMG comparison

After analysis, and by grouping the categories into two, mild or moderate CTS and severe CTS, there was a statistically significant difference between ENMG grade and ultrasound colour Doppler signal (p < 0.001) between the group with severe CTS and the group with non-severe CTS (Table 5).

**Table 5:** Colour Doppler on ultrasound in the diagnosis of CTS severity compared to ENMG *Values expressed as percentage (%) CTS: carpal tunnel syndrome; ENMG: electroneuromyography

Color Doppler result	Mild or moderate CTS	Severe CTS	p-value
Colour Doppler positive*	25	75	< 0.001
Colour Doppler negative*	70.3	24.8	

## Discussion

Our study concludes that colour Doppler obtained by ultrasound can predict the severity of CTS, demonstrating a difference between the severity of CTS and the presence of intraneural hypervascularisation of the median nerve on colour Doppler. Median nerve ultrasound examination was recommended in the evaluation of CTS patients in the 1990 guidelines, as a complement to ENMG [16]. Its role in the diagnosis of CTS has been widely demonstrated in several studies, which have shown a correlation between CSA and the positive diagnosis and severity of CTS [17-19], with the possibility of assessing the degree of median nerve distress: mild between 10 sq mm and 12.9 sq mm, moderate between 13 sq mm​​​​​​​ and 14.9 sq mm​​​​​​​, and severe if greater than 15 sq mm​​​​​​​ [16]. However, some studies suggest that CSA does not predict CTS severity [20-22]. This indicates that while CSA is an excellent screening tool for CTS, it cannot be used to grade CTS severity.

Doppler colour has the advantage of being speedy, cost-effective, convenient, and non-invasive, allowing for early detection of CTS [23] and even post-surgical follow-up in cases of incomplete resection, causing symptom recurrence [24]. However, Doppler colour does not allow for the visualisation of morphological disturbances, and its accuracy depends on the operator’s experience [25].

The effectiveness of colour Doppler in the positive diagnosis of CTS has been demonstrated in several studies, which have shown hypervascularization in patients with CTS [17, 25-27], with a specificity of 80% and a sensitivity of 85% [27], median nerve hyperaemia Is related to blockade of venous outflow during early compression in CTS, leading to compensatory dilation of perineural veins, a decrease in arterial supply, ischemic injury, and edema [28]. Many studies have suggested that this increased vascularity, associated with direct nerve pressure, may be linked to disease severity. However, the association between intraneuronal hypervascularisation of the median nerve and the severity of CTS remains unclear. Studies on this issue have shown conflicting results. Two studies showed that intraneuronal median nerve vasculature was associated with CTS severity [25, 29]. Others did not find a significant correlation [21, 30-31], concluding that colour Doppler is not able to determine the severity of CTS. A study conducted by Nam et al. showed that none of the ultrasound parameters, including colour Doppler and pulsed Doppler, differentiated between stages of CTS severity [32]. In another study conducted by Ozcan et al., it was shown that the CSA of the median nerve, its flattening ratio, flexor retinaculum bowing, and intraneural hypervascularization were significantly correlated with the degree of severity of CTS (p < 0.001) [26]. An inverse relationship may exist between intraneural vascular flow in the median nerve and increasing severity of CTS based on nerve conduction results [27].

Limitations of our study

The small size of our sample is the main weakness of our study, along with the use of colour Doppler without quantisation and motion artefacts.

## Conclusions

Our study concludes that colour Doppler ultrasound can predict the severity of CTS, showing a difference between the severity of CTS and the presence of intraneural hypervascularisation of the median nerve on colour Doppler. It can be used in routine practice for assessing the severity of CTS. We believe that colour Doppler should be a part of the ultrasound evaluation of CTS. Further studies will be needed to evaluate the sensitivity and specificity of colour Doppler ultrasound, including Doppler quantification.

## References

[1] Atroshi I, Gummesson C, Johnsson R, Ornstein E, Ranstam J, Rosén I (1999). Prevalence of carpal tunnel syndrome in a general population. JAMA.

[2] Gervasio A, Stelitano C, Bollani P, Giardini A, Vanzetti E, Ferrari M (2020). Carpal tunnel sonography. J Ultrasound.

[3] Jablecki CK, Andary MT, Floeter MK, Miller RG, Quartly CA, Vennix MJ, Wilson JR (2002). Practice parameter: Electrodiagnostic studies in carpal tunnel syndrome. Report of the American Association of Electrodiagnostic Medicine, American Academy of Neurology, and the American Academy of Physical Medicine and Rehabilitation. Neurology.

[4] Lew HL, Date ES, Pan SS, Wu P, Ware PF, Kingery WS (2005). Sensitivity, specificity, and variability of nerve conduction velocity measurements in carpal tunnel syndrome. Arch Phys Med Rehabil.

[5] Atroshi I, Gummesson C, Johnsson R, Ornstein E (2003). Diagnostic properties of nerve conduction tests in population-based carpal tunnel syndrome. BMC Musculoskelet Disord.

[6] Chompoopong P, Preston DC (2021). Neuromuscular ultrasound findings in carpal tunnel syndrome with symptoms mainly in the nondominant hand. Muscle Nerve.

[7] Jain S, Visser LH, Praveen TL (2009). High-resolution sonography: a new technique to detect nerve damage in leprosy. PLoS Negl Trop Dis.

[8] Vijayan J, Chan YC, Therimadasamy A, Wilder-Smith EP (2015). Role of combined B-mode and Doppler sonography in evaluating neurolymphomatosis. Neurology.

[9] Goedee HS, Brekelmans GJ, Visser LH (2014). Multifocal enlargement and increased vascularization of peripheral nerves detected by sonography in CIDP: a pilot study. Clin Neurophysiol.

[10] Frijlink DW, Brekelmans GJ, Visser LH (2013). Increased nerve vascularization detected by color Doppler sonography in patients with ulnar neuropathy at the elbow indicates axonal damage. Muscle Nerve.

[11] Fowler JR, Gaughan JP, Ilyas AM (2011). The sensitivity and specificity of ultrasound for the diagnosis of carpal tunnel syndrome: a meta-analysis. Clin Orthop Relat Res.

[12] Sheen S, Ahmed A, Raiford ME (2024). Association between electrodiagnosis and neuromuscular ultrasound in the diagnosis and assessment of severity of carpal tunnel syndrome. PM R.

[13] Ozsoy-Unubol T, Bahar-Ozdemir Y, Yagci I (2020). Diagnosis and grading of carpal tunnel syndrome with quantitative ultrasound: is it possible?. J Clin Neurosci.

[14] Stevens JC (1997). AAEM minimonograph #26: the electrodiagnosis of carpal tunnel syndrome. American Association of Electrodiagnostic Medicine. Muscle Nerve.

[15] Toth F, Kiss E, Marafko C, Nemes J, Hegedus Z (2021). The clinical value of the self-administered Kamath and Stothard’s questionnaire in the diagnostics of carpal tunnel syndrome. Open J Thera Rehab.

[16] Cartwright MS, Hobson-Webb LD, Boon AJ (2012). Evidence-based guideline: neuromuscular ultrasound for the diagnosis of carpal tunnel syndrome. Muscle Nerve.

[17] Vanderschueren GA, Meys VE, Beekman R (2014). Doppler sonography for the diagnosis of carpal tunnel syndrome: a critical review. Muscle Nerve.

[18] Akcar N, Ozkan S, Mehmetoglu O, Calisir C, Adapinar B (2010). Value of power Doppler and gray-scale US in the diagnosis of carpal tunnel syndrome: contribution of cross-sectional area just before the tunnel inlet as compared with the cross-sectional area at the tunnel. Korean J Radiol.

[19] Ikeda M, Okada M, Toyama M, Uemura T, Takamatsu K, Nakamura H (2017). Comparison of median nerve cross-sectional area on 3-T MRI in patients with carpal tunnel syndrome. Orthopedics.

[20] Karahan AY, Arslan S, Ordahan B, Bakdik S, Ekiz T (2018). Superb microvascular imaging of the median nerve in carpal tunnel syndrome: an electrodiagnostic and ultrasonographic study. J Ultrasound Med.

[21] Bayrak IK, Bayrak AO, Tilki HE, Nural MS, Sunter T (2007). Ultrasonography in carpal tunnel syndrome: comparison with electrophysiological stage and motor unit number estimate. Muscle Nerve.

[22] Kwon HK, Kang HJ, Byun CW, Yoon JS, Kang CH, Pyun SB (2014). Correlation between ultrasonography findings and electrodiagnostic severity in carpal tunnel syndrome: 3D ultrasonography. J Clin Neurol.

[23] Mallouhi A, Pülzl P, Trieb T, Piza H, Bodner G (2006). Predictors of carpal tunnel syndrome: accuracy of gray-scale and color Doppler sonography. AJR Am J Roentgenol.

[24] Bianchi S, Montet X, Martinoli C, Bonvin F, Fasel J (2004). High-resolution sonography of compressive neuropathies of the wrist. J Clin Ultrasound.

[25] Mohammadi A, Ghasemi-Rad M, Mladkova-Suchy N, Ansari S (2012). Correlation between the severity of carpal tunnel syndrome and color Doppler sonography findings. AJR Am J Roentgenol.

[26] Ozcan HN, Kara M, Ozcan F, Bostanoglu S, Karademir MA, Erkin G, Ozçakar L (2011). Dynamic Doppler evaluation of the radial and ulnar arteries in patients with carpal tunnel syndrome. AJR Am J Roentgenol.

[27] Ghasemi-Esfe AR, Morteza A, Khalilzadeh O, Mazloumi M, Ghasemi-Esfe M, Rahmani M (2012). Color Doppler ultrasound for evaluation of vasomotor activity in patients with carpal tunnel syndrome. Skeletal Radiol.

[28] Werner RA, Andary M (2002). Carpal tunnel syndrome: pathophysiology and clinical neurophysiology. Clin Neurophysiol.

[29] Ghasemi-Esfe AR, Khalilzadeh O, Vaziri-Bozorg SM, Jajroudi M, Shakiba M, Mazloumi M, Rahmani M (2011). Color and power Doppler US for diagnosing carpal tunnel syndrome and determining its severity: a quantitative image processing method. Radiology.

[30] Öztürk GT, Malas FÜ, Yildizgören MT (2015). Ultrasonographic assessment of the femoral cartilage thickness in patients with pes planus: a multicenter study by TURK-MUSCULUS. Am J Phys Med Rehabil.

[31] Yoshii Y, Tanaka T, Ishii T (2016). Correlations of median nerve area, strain, and nerve conduction in carpal tunnel syndrome patients. Hand (N Y).

[32] Nam K, Peterson SM, Wessner CE, Machado P, Forsberg F (2021). Diagnosis of carpal tunnel syndrome using shear wave elastography and high-frequency ultrasound imaging. Acad Radiol.

